# Factors associated with seroconversion to the major piroplasm surface protein of the bovine haemoparasite *Theileria orientalis*

**DOI:** 10.1186/s13071-016-1395-7

**Published:** 2016-02-25

**Authors:** Cheryl Jenkins, Daniel R. Bogema

**Affiliations:** NSW Department of Primary Industries, Elizabeth Macarthur Agricultural Institute, Menangle, NSW 2568 Australia; The ithree institute, University of Technology, Sydney, Ultimo, NSW 2007 Australia

**Keywords:** *Theileria orientalis*, Major piroplasm surface protein (MPSP), Seroconversion, Anaemia, Genotype

## Abstract

**Background:**

Bovine theileriosis caused by *Theileria orientalis* is an emerging disease of cattle in the Asia-Pacific region where it causes a significant economic burden to meat and milk production. While host immunological responses to the lymphocyte-transforming species of *Theileria*, *T. parva* and *T. annulata*, have been well studied, little is known about the immune response to this non-transforming species.

**Methods:**

We developed a recombinant antigen ELISA based on the major piroplasm surface protein (MPSP) of *T. orientalis* and investigated whether seroconversion to the MPSP was associated with clinical factors (anaemia), parasite burden and parasite genotype. We also examined the dynamics of seroconversion in animals acutely infected with *T. orientalis*.

**Results:**

In cattle testing qPCR positive for *T. orientalis*, seroconversion was more frequent in anaemic compared to normal cattle (*P* < 0.0001). The ELISA ratio (ER) was highly correlated with total parasite burden as measured by qPCR (*r* = 0.69; *P* < 0.0001); however when loads of individual genotypes of the parasite were examined, only the pathogenic Ikeda genotype was highly correlated with ER. Conversely, seroconversion was less frequently detected in the presence of benign *T. orientalis* genotypes. Temporal measurement of the serological response, parasite burden and packed cell volume (PCV) in acutely infected animals revealed that seroconversion to the MPSP occurs within 2-3 weeks of the initial qPCR detection of the parasite and coincides with a peak in infection intensity and a declining PCV.

**Conclusion:**

Whether the serological response to the MPSP is immunoprotective against re-infection or recrudescence requires further investigation; however the MPSP represents a promising target for a subunit vaccine given that genetic variability within the MPSP results in differential pathogenicity of *T. orientalis*.

## Background

*Theileria orientalis* is an emerging apicomplexan pathogen of cattle in the Asia-Pacific region. Previously described as benign, this haemoprotozoan is now recognised as a disease of production cattle causing anaemia and ill-thrift. Mortalities of up to 5 %, particularly in pregnant heifers and calves, have been reported and the disease is also commonly associated with late term abortion. The bush tick *Haemaphysalis longicornis* is recognised as the vector for disease transmission [1, 2], and the geographic distribution of recent bovine theileriosis cases in Australia and New Zealand closely follow the known range of this species [2, 3].

Many recent studies have focussed on identification and differentiation of various genotypes of *T. orientalis*. These genotypes are defined based on sequence variations in the gene encoding the major piroplasm surface protein (MPSP), an immunodominant antigen expressed during both sporozoite and piroplasm phases of the *T. orientalis* life-cycle [4]. While eleven genotypes of *T. orientalis* have been identified globally (Types 1-8 and N1-N3) [5], disease outbreaks in cattle have been largely limited to Type 2 (Ikeda genotype) [6–9], with only occasional reports of clinical cases linked to other genotypes [10, 11]. Genotype 3 (Buffeli) [6, 7, 12] and its phylogenetic relative, Type 5 [7] have also been identified in Australian cattle, but these genotypes have not been associated with clinical disease and are considered benign.

Like other apicomplexans [13, 14], *T. orientalis* infection most frequently presents as a mixture of genotypes, which likely facilitates evasion of the host immune system [15–17]. Indeed, infected cattle appear to retain the parasite for lengthy periods, perhaps for life [15]. Subclinical infections with *T. orientalis* including the Ikeda genotype are common [18] however; the immune mechanisms responsible for disease resistance are poorly understood. Naïve cattle introduced to areas where the disease is enzootic, as well as stressed, immunocompromised, pregnant or lactating animals are most at risk of developing clinical disease [6, 12, 19], while cattle in *T. orientalis*-endemic areas appear to develop a degree of resistance to disease. It is unclear whether the host develops a humoral response against the parasite prior to the intra-erythrocytic phase of the parasite’s life-cycle, or whether the immune response is largely cell-mediated. Prior studies on *T. annulata* and *T. parva* suggest that responses against these parasites are largely cell-mediated [20, 21], however these organisms represent transforming theilerias which cause a tumour-like lymphocytic proliferation [22] not observed in *T. orientalis* infection.

In *T. orientalis*, the MPSP is highly expressed during both the sporozoite [4] and piroplasm [23] phases of the parasite’s life-cycle and is believed to mediate entry into bovine erythrocytes *via* interactions with heparin-like compounds on the host cell surface [24]. Immunoblots using sera from infected animals indicate that the MPSP is strongly recognised by host IgG and that immunisation of cattle with MPSP is at least partially protective against *T. orientalis* [25]. Nonetheless, immune response to this antigen has not yet been quantified in cattle using ELISA, although this method was found to be a sensitive means of detecting *T. orientalis* infection in water buffalo [26].

In this study, we developed a recombinant MPSP ELISA to measure bovine IgG response to this major surface antigen and correlate this response with clinical disease, parasite genotype and infection intensity.

## Methods

### Samples

A total of 430 EDTA blood samples and their matching sera were analysed in this study. Of these, 280 pairs of samples were collected by private and district veterinarians from 21cattle herds from the states of New South Wales (NSW) and Queensland (QLD), Australia. These samples were collected as part of routine clinical investigations into the significance of the *Theileria orientalis* genotypes in Australian cattle [12] or were submitted to the Elizabeth Macarthur Agricultural Institute as clinical samples from suspect theileriosis cases [6]. A further 60 pairs of samples served as negative controls; 50 of these were collected from cattle herds located in regions known to be free of *T. orientalis* and a further 10 pairs of samples were derived from cattle infected with *Babesia bigemina* (*n* = 3), *Babesia bovis* (*n* = 4) or *Anaplasma centrale* (*n* = 3). The remaining pairs of samples (*n* = 90) were collected from a single herd of 10 naïve animals (2 year- old Ayrshire heifers) that had been introduced to a property on the mid-coast of NSW with a history of clinical theileriosis cases [16]. Briefly, EDTA blood was collected from each animal immediately upon introduction to the affected property, and approximately weekly thereafter for a period of 76 days [16]. Sera were prepared from clotted blood or where clotted blood was unavailable; plasma was prepared from blood containing anti-coagulant. These samples were used for a temporal study examining seroconversion to the *T. orientalis* MPSP.

### Packed cell volume and blood films

Of the EDTA blood samples collected, 256 were examined for packed cell volume and 376 were examined *via* blood film, as described previously [12, 16]. Animals with a PCV < 24 % were considered anaemic.

### Cloning and expression of the T. orientalis MPSP genes

MPSP genes amplified from each of the *T. orientalis* Ikeda, Chitose and Buffeli MPSP types which had been cloned into the Champion pET100 D-TOPO expression vector (Invitrogen, Carlsbad, California, USA) [27] were used to express recombinant antigen for Western blotting and ELISA assays. The recombinant proteins were purified using nickel affinity chromatography and dialysed as previously described [28].

### Western blotting

Western blots of recombinant MPSP antigen derived from three genotypes of *T. orientalis* (Ikeda, Chitose and Buffeli; predicted sizes 34-35 kDa) were screened with antisera from cattle previously confirmed PCR positive for a single MPSP genotype [6, 12]. The blots were performed as described previously [29], except that 1 μg of each MPSP protein was blotted against a 1:100 dilution of each primary antiserum. The secondary antibody was a 1:20 000 dilution of alkaline phosphatase-conjugated anti-bovine (Sigma Aldrich, St Louis, Missouri USA; Catalogue number A7554). Blots were developed using NBT/BCIP substrate (Sigma, Catalogue number 11697471001).

### ELISA

The recombinant MPSP ELISA was performed using a cocktail of Ikeda, Chitose and Buffeli MPSP antigen at equimolar concentrations as described previously [27], except that 0.75 μg of total antigen (ie: 0.25 μg of each antigen) was used to coat the ELISA plate wells. A 1 × Blocking Buffer solution (Cat. No. B6429; Sigma, St. Louis, MO, USA) was used to block the plates and as an antibody diluent. All washes were performed as described previously [27] but using phosphate buffered saline containing 0.05 % Tween 20 (PBST). Results were expressed as an ELISA ratio (ER: mean OD_610_ test serum/mean OD_610_ of the negative control serum). Sera with an ER < 2 were considered negative, an ER ≥ 2 as positive.

### DNA extraction and quantitative PCR (qPCR)

DNA extractions were performed using the DNeasy Blood and Tissue DNA extraction kit (Qiagen). All DNA extractions and quantitative PCRs for *T. orientalis* (universal) and the genotypes Ikeda and Chitose (UIC qPCR) were performed as previously described [30].

### Statistical analysis

Associations between PCV and ELISA ratio and parasite load and ELISA ratio were analysed using Spearman’s rank correlation (r) for non-parametric data within Prism 4 (GraphPad Software Inc., La Jolla, California, USA). Fisher’s exact test (GraphPadQuickCalcs:http://www.graphpad.com/quickcalcs/) was used to determine whether there was an association between serological status and clinical presentation (anaemic *vs* normal). The probability that infection level was related to serological status was determined using the Freeman-Halton extension of the Fisher’s exact test *via* the VassarStats website (http://vassarstats.net/fisher2x3.html). *P* values calculated for both tests are two-tailed.

## Results

### Immunological cross-reactivity between genotype MPSPs

**Fig. 1:**
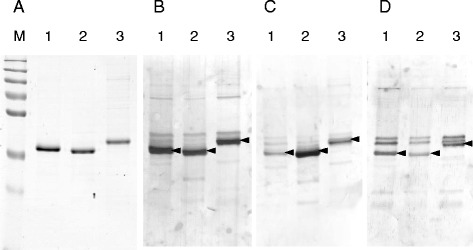
Western blot analysis of recombinant MPSP antigens from the Ikeda (Lane 1), Chitose (Lane 2) and Buffeli (Lane 3) genotypes of *T. orientalis*. One microgram of each recombinant protein was run on an 1D SDS-PAGE gel and stained (**a**) or blotted against sera from animals infected with the Ikeda (**b**), Chitose (**c**) and Buffeli (**d**) genotypes of *T. orientalis*. Immunoreactive MPSP proteins are indicated with arrowheads. *M* = molecular weight marker (sizes 250, 130, 100, 75, 55, 35 and 25 kDa).

While the MPSP gene is the most commonly used marker to discriminate between the various *T. orientalis* genotypes, the proteins encoded by these genes display a high degree of sequence conservation. Studies using native and recombinant MPSP proteins have shown conflicting results with respect to serological cross-reactivity between genotype MPSPs, with native [31] but not recombinant [32, 33] antigen being serologically distinct amongst genotypes. Nonetheless, recombinant MPSPs can be discriminated using monoclonal antibodies against specific epitopes [32], while post-translational modifications are believed to be responsible for serological discrimination amongst native MPSPs [33]. Western blotting of recombinant MPSP antigen from the Ikeda, Chitose and Buffeli genotypes of *T. orientalis* was undertaken to determine whether there was serological cross-reactivity between the genotype MPSPs generated in this study. Sera from animals infected with individual genotypes of the parasite (Ikeda, Chitose or Buffeli as determined by qPCR) displayed cross-reactivity with all three MPSP types (representative blots are shown in Fig. 1). However, some variability in the reactivity of serum in detecting their specific targets was observed based on the intensity of the bands detected (Fig. 1); therefore the MPSP antigens were pooled for subsequent use in the ELISA assay.

### Specificity and sensitivity of the MPSP ELISA

The specificity and sensitivity of the MPSP ELISA compared to blood film examination and qPCR was assessed using 376 pairs of EDTA bloods and sera for which all three assays had been conducted. PCR assays are considered a gold standard for *T. orientalis* detection, and consistent with this, the UIC qPCR detected the highest number of positive samples (262/376). Using qPCR as a gold standard for comparison, the MPSP ELISA had a sensitivity of 68.7 % and a specificity of 99.1 %, while blood film examination had a sensitivity of 62.2 % and a specificity of 97.4 %. Animals that were PCR positive but ELISA negative either may have not yet seroconverted to the MPSP antigen or did not elicit a strong enough humoral response to enable detection. Thus, while ELISA was found to be more sensitive and specific for *T. orientalis* detection than blood film, as previously demonstrated for *T. orientalis* detection in buffalo [26], PCR-based methods remain the most sensitive option for parasite detection.

### MPSP seropositivity is associated with anaemia

**Fig. 2:**
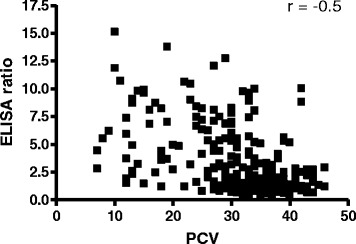
The ELISA ratios (ER) of individual animal sera are negatively correlated with the packed cell volume (PCV) of whole blood (*r* = -0.5).

Packed cell volume (PCV) was used as a measure of clinical status and was compared to the corresponding serological data from individual animals. Of the animals with a PCV in the normal range (PCV ≥ 24), 55 % were seronegative, while the majority of animals (89 %) with anaemia were MPSP seropositive (Table 1). Fisher’s exact test indicated that the association between MPSP seropositivity and anaemia was significant (*P* < 0.0001). When the ELISA ratios of individual animals were plotted against the corresponding PCVs a statistically significant moderate and negative correlation (*r* = -0.5) was observed (Fig. 2).

**Table 1:** Frequency of anaemic versus normal animals in relation to MPSP serological status

	Anaemic (*n* = 36)	Normal (*n* = 220)
MPSP seropositive	32 (89 %)	98 (45 %)
MPSP seronegative	4 (11 %)	122 (55 %)

*P* < 0.0001

### MPSP seropositivity is associated with total parasite load and the Ikeda genotype

**Fig. 3:**
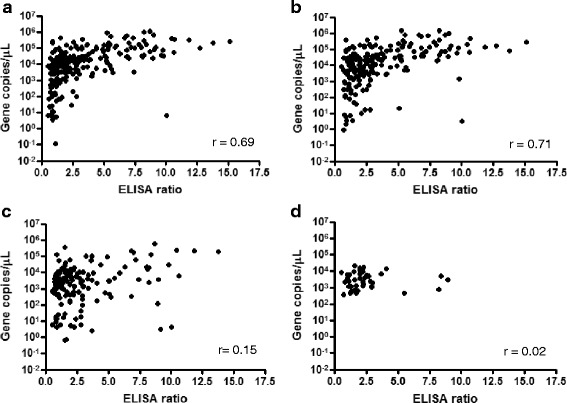
(**a**) The total parasite load (gene copies/μL of blood) as measured by qPCR is positively correlated with the ELISA ratio (ER). Levels of the Ikeda genotype are strongly correlated with ER (**b**), while the levels of the Chitose genotype are only weakly correlated (**c**). No correlation was observed between levels of the Buffeli genotype and ER (**d**).

The parasite load within the blood of individual animals was measured using the universal (*T. orientalis*) component of a multiplex quantitative PCR assay [30]. Because *T. orientalis* infections often present as a mixture of genotypes, the relative quantities of the genotypes previously reported to be associated with clinical disease (Ikeda and Chitose genotypes) were also measured using the Ikeda and Chitose-specific components of the multiplex qPCR [30]. A Buffeli-specific qPCR was also used to measure the quantities of this genotype [16]. The relationship between the MPSP ELISA ratio and the total number of *T. orientalis* gene copies per μL of blood (GC/μL) or those of the individual *T. orientalis* genotypes is shown in Fig. 3. We observed a strong and significant positive correlation between the load of *T. orientalis* and the MPSP ELISA ratio (*r* = 0.69, *P* < 0.0001; Fig. 3a). Comparison of individual genotype load with MPSP ELISA ratio revealed a strong positive correlation for the Ikeda genotype only (*r* = 0.71, *P* < 0.0001; Fig. 3b). There was a weak but significant correlation (*r* = 0.15) between the MPSP ELISA ratio and the Chitose genotype (*p* = 0.02; Fig. 3c). No relationship was observed between the quantity of the Buffeli genotype and MPSP ELISA ratio (*r* = 0.02, *p* = 0.9; Fig. 3d).

The *T. orientalis* qPCR data were divided into three categories of infection intensity (low medium and high) based on previously established clinical thresholds [30]. Only 35 % of animals with infections in the low category (<15 000 MPSP GC/μL), which typically represent subclinical carriers, were MPSP seropositive. Animals in the moderate (>15 000 but <300 000 MPSP GC/μL) category, which usually represent recovering, in-contact or clinically-affected animals, had a much higher rate of MPSP seropositivity at 75 %. In a previous study, 92 % of animals in the high infection intensity range were shown to have one or more signs of clinical theileriosis [30]; 83 % of animals in this category tested seropositive (Table 2).

**Table 2:** Relationship between infection level and serological status

	Infection level*		
	High (*n* = 12)	Moderate (*n* = 106)	Low (*n* = 110)
MPSP seropositive	11 (83 %)	80 (75 %)	39 (35 %)
MPSP seronegative	1 (17 %)	26 (25 %)	71 (65 %)

*P* < 0.0001

*Infection levels are as defined in [30]. Low: <15 000 gene copies/μL blood; Moderate: >15 000 but <300 000 gene copies/μL blood; High: >300 000 gene copies/μL blood.

### Temporal dynamics of MPSP seroconversion

**Fig. 4:**
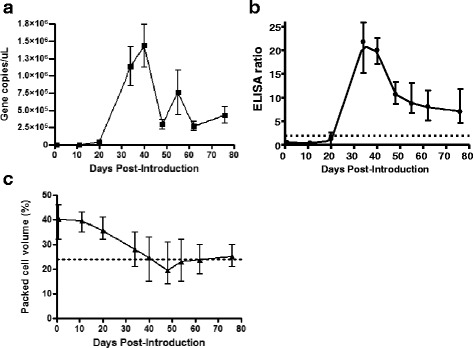
Temporal dynamics of *T. orientalis* infection as determined by qPCR (**a**), MPSP serological response (**b**) and packed cell volume (**c**) in 10 naïve animals introduced to a property with a history of clinical theileriosis. Parasite load and serological response peaked between Day 34 and 40 post introduction (**a**& **b**), after which time the antibody titre declined sharply but remained above the ELISA positive cut-off (dotted line). The PCV of the infected animals began to decline at Day 20 post-introduction (**c**), with animals falling into the anaemic range between Day 40 and Day 48 post-introduction. All graphs show the mean and range of data from the 10 animals.

We examined the temporal dynamics of seroconversion to the *T. orientalis* MPSP antigen by testing paired sera and EDTA blood from 10 naïve cattle that were introduced to a property with a known history of clinical theileriosis cases. qPCR was used to monitor the progress of infection and the serological response to the MPSP antigen was measured by MPSP ELISA. The PCV of each animal was also tested. All animals became rapidly infected with *T. orientalis* following introduction to the affected property, with 7 animals testing qPCR positive by Day 11 post-introduction and the remaining 3 animals testing positive by Day 20. The average peak in parasite load was at Day 40 post-introduction (Fig. 4a). In contrast, a positive serological response to the MPSP antigen was detected in only 1 animal at Day 20 post-introduction (this animal was qPCR positive at Day 11); however all 10 animals had seroconverted by Day 34 (Fig. 4b). The peak serological response occurred between Days 34 and 40 and preceded a sharp drop in parasite load between Days 40 and 48. The MPSP serological response declined steadily from Day 40 until the end of the sampling period (Day 76); however all animals remained in the positive range over this period. The average PCV of the cattle began to drop on Day 20, shortly after or coinciding with the onset of infection, and reaching a trough between Days 40 and 54 (Fig. 4c). All animals except one entered the anaemic range following the peak in infection intensity and the MPSP antibody response. In 9/10 cows, the decline in PCV commenced prior to the animals returning their first seropositive result; in the remaining animal, the decline in PCV coincided with the first seropositive result on Day 20; however at this time the animal was only just over the positive ER threshold of 2.

## Discussion

The drivers of the host immunological response to *T. orientalis* infection are poorly studied but require investigation if effective vaccines are to be developed against this parasite in the future. Here we examined factors associated with stimulation of a humoral response to a major surface protein (MPSP) of *T. orientalis* which is expressed during both the merozoite (piroplasm) and sporozoite life-cycle phases of the parasite. The serological status of *T. orientalis*-infected animals is of interest given a potential role for humoral immunity in the host response to infection. While humoral responses to intracellular pathogens have traditionally been considered of less importance than cell-mediated responses, there are many recent examples where antibodies have been found to afford protection by acting in concert with the cellular immune system or by neutralising extracellular phases of the pathogen prior to gaining cell entry [34].

While the host response to infection in the transforming *Theileria* spp. (*T. parva* and *T. annulata*) appears to be largely mediated by cytotoxic T cell responses against macroschizont-infected lymphoblasts [35]; *T. orientalis* lacks the ability to transform leukocytes. In contrast, the pathogenic phase of *T. orientalis* is the intraerythrocytic phase, with the major symptoms of disease resulting from erythrocyte destruction and subsequent anaemia. In this study we found a significant association between MPSP seropositivity and anaemia with 89 % of anaemic animals seroconverting to the MPSP antigen. Conversely, only 45 % of animals with PCVs in the normal range had seroconverted. While seroconversion to *T. parva* shows a small but significant inverse association with PCV, the association between anaemia and seroconversion to the tick fever parasite, *Babesia bigemina* is much more marked [36]. Like *T. orientalis*, *B. bigemina* is a non-transforming member of the Piroplasmida, exerting its major pathogenic effects *via* its intraerythrocytic phase [37]. Thus, it appears that in both *B. bigemina* and *T. orientalis* infection, seroconversion is linked to erythrocyte destruction. This observation was consistent with data from our temporal study in which 10 naïve cows became acutely infected with *T. orientalis*. In these animals, seroconversion to the MPSP antigen had already occurred (Day 34) by the time the parasite load peaked (Day 40). Furthermore, while the animals did not become anaemic until approximately 10 days after parasite load and serological response had peaked, the onset of the decline in PCV commenced very early in the time course (Day 20 on average). In addition, 9 of 10 animals were seronegative on Day 20, despite the fact that the majority of animals tested qPCR positive for *T. orientalis* by Day 11. Furthermore, in a prior study using a *T. orientalis* ELISA based on crude whole antigen, the onset of the serological response coincided with the appearance of the piroplasm phase in red blood cells [38]. Together, these data suggest that seroconversion may occur as a consequence of the release of free parasites/parasite antigen from lysed erythrocytes rather than in response to the initial inoculation of sporozoites from the tick. Indeed prior studies on *Theileria* spp. indicate that the sporozoites become rapidly internalised (within 3 minutes) of cell attachment [39].

In this study, we revealed a strong, positive and significant correlation between the MPSP ELISA ratio and the total parasite load as determined by qPCR. Furthermore, when the levels of the individual *T. orientalis* genotypes were examined, the correlation was strongest (*r* = 0.71) with the load of the *T. orientalis* Ikeda genotype. The Ikeda genotype has been linked to many clinical outbreaks of bovine theileriosis in the Asia-Pacific region [7–9, 12] and is considered the major pathogenic genotype of this parasite. In contrast the levels of the Chitose and Buffeli genotypes, which are very rarely associated with disease, showed only a weak or no correlation with the MPSP ELISA ratio respectively. We demonstrated in a prior study that the Ikeda genotype of *T. orientalis* is frequently associated with high infection intensities and furthermore, that infection intensity is negatively correlated with PCV [30]. The decline in PCV resulting from *T. orientalis* Ikeda infection reflects enhanced erythrocyte destruction in the presence of this genotype and consequently, the increased likelihood of a serological response associated with infection by this genotype.

Results from our temporal study indicated that animals acutely infected with *T. orientalis* seroconverted 2-3 wk after the parasite was detectable via qPCR (34 days post-introduction to the affected herd). This is directly comparable to a study on the tick fever parasites, *Babesia bovis* and *B. bigemina*, in which animals introduced to a property with ticks harbouring these parasites all seroconverted within 35 days, coinciding with a decline in their PCVs [40]. Nonetheless, tick-borne parasites vary in their ability to induce a sustained serological response in their hosts. Animals acutely infected with *T. parva* may die prior to seroconversion; however surviving animals tend to produce a long-lasting serological response and are generally immune to re-infection thereafter [41]. While the longevity of the serological response to *Babesia* spp. is variable, seropositivity is often sustained for between 18 months and 6 years [37, 40, 42]. In contrast, *Theileria mutans* induces only short-term serological responses [41]. In the case of *T. orientalis*, we demonstrated that the antibody titre declines steeply (along with the parasite burden) following acute infection; however in all animals examined, the MPSP serological response plateaued within the positive range for the remainder of the study period (76 days). Whether the serological response is sustained over the longer term is yet to be determined.

While cell-mediated responses are central to protection from re-infection with the transforming theilerias [5, 21, 43] the pathogenesis of *T. orientalis* more closely resembles that of the non-transforming piroplasmids, *B. bovis* and *B. bigemina. Bigemina bovis* infection is concomitant: animals remain persistently infected with the parasite, but resist disease relapse [44]. While *T. orientalis* infections are known to persist long-term [15], it is currently unclear whether acutely infected animals become refractory to subsequent disease. In *B. bovis* infection, immune protection appears to involve a combination of innate and cell-mediated immunity and neutralising antibody [44]. The spleen is central to the host response to acute disease and removes parasitised erythrocytes from circulation, while subsequent stimulation of CD4+ T lymphocytes and the production of neutralising antibody appear to be responsible for subsequent adaptive immunity to the parasite. The spleen is evidently of similar importance in responding to acute theileriosis caused by *T. orientalis*, with splenomegaly a common finding on necropsy [19] and splenectomy inducing high parasitaemias in infected animals [45]. Indeed, splenic clearance of parasitised erythrocytes is most likely responsible for the declines in PCV observed in acutely affected animals in this study and the spleen may also be important in the generation of the serological response via the white pulp.

In *B. bovis* infection, antibodies are believed to be most important in neutralising extracellular phases of the parasite including the sporozoite and merozoite phases, and in capturing erythrocytes expressing parasite antigen on the cell surface [44]. In some cases, immunity to *Babesia* infection only appears to occur following seroconversion [37, 46, 47]. Whether the humoral response to the *T. orientalis* MPSP protects against recrudescence or re-infection with new *T. orientalis* strains remains unclear, but is worthy of further investigation. Indeed, one study suggested that passive transfer of monoclonal antibodies raised against MPSP epitopes had an immunoprotective effect on calves challenged with *T. orientalis* [48]. Similarly, calves vaccinated with either recombinant MPSP antigen or peptides derived from the MPSP antigen showed reduced parasitaemias and an absence of clinical signs of disease relative to control calves following experimental challenge [25]. Further investigation of the *T. orientalis* MPSP as a potential candidate for a subunit vaccine is worthwhile due to the promising results from these initial investigations. While live vaccines are generally favoured for the prevention of apicomplexan infections due to high levels of genetic variation within these organisms, *T. orientalis* may represent a unique case given that a single MPSP genotype (Ikeda) is correlated with the majority of clinical outbreaks.

## Conclusions

We demonstrate that seroconversion to the *T. orientalis* MPSP is significantly associated with host anaemia, parasite load and parasite genotype. Acutely infected animals seroconvert quickly (within 2-3 weeks post-infection) and the humoral response to the parasite is maintained for at least 10 weeks. The *T. orientalis* MPSP should be further considered as a vaccine target for the prevention of bovine theileriosis in the Asia-Pacific.
